# Accuracy of self-reported history of autoimmune disease: A pilot study

**DOI:** 10.1371/journal.pone.0216526

**Published:** 2019-05-29

**Authors:** Julia A. O'Rourke, Caitlin Ravichandran, Yamini J. Howe, Jennifer E. Mullett, Christopher J. Keary, Sara B. Golas, Amrita R. Hureau, Morgan McCormick, Jeanhee Chung, Noel R. Rose, Christopher J. McDougle

**Affiliations:** 1 Lurie Center for Autism, Lexington, MA, United States of America; 2 Massachusetts General Hospital, Boston, MA, United States of America; 3 Harvard Medical School, Boston, MA, United States of America; 4 Program for Neuropsychiatric Research, McLean Hospital, Belmont, MA, United States of America; 5 Department of Psychiatry, Indiana University School of Medicine, Indianapolis, IN, United States of America; 6 Brigham and Women’s Hospital, Boston, MA, United States of America; University of Wyoming College of Health Sciences, UNITED STATES

## Abstract

Research associating the increased prevalence of familial autoimmunity with neuropsychiatric disorders is reliant upon the ascertainment of history of autoimmune diseases from relatives. To characterize the accuracy of self-report, we compared self-reported diagnoses of 18 autoimmune diseases using an online self-report questionnaire to the electronic medical record (EMR) diagnoses in 1,013 adult (age 18–70 years) patients of a primary care clinic. For the 11 diseases meeting our threshold observed prevalence, we estimated sensitivity, specificity, positive predictive value (PPV), and negative predictive value (NPV) for self-reported diagnoses under the assumption that EMR-based diagnoses were accurate. Six diseases out of 11 had either sensitivity or PPV below 50%, with the lowest PPV for dermatological and endocrinological diseases. Common errors included incorrectly self-reporting type 2 diabetes mellitus (DM), when type 1 DM was indicated by the EMR, and reporting rheumatoid arthritis when osteoarthritis was indicated by the EMR. Results suggest that ascertainment of familial autoimmunity through self-report contributes to inconsistencies and inaccuracies in studies of autoimmune disease history and that future studies would benefit from incorporating EMR review and biological measures.

## Introduction

Immune dysfunction contributes to the pathophysiology of a number of neuropsychiatric disorders.[1] At the clinical descriptive level, studies have found a higher prevalence of familial autoimmunity in individuals with different neuropsychiatric disorders, including autism spectrum disorder (ASD),[2, 3] attention-deficit/hyperactivity disorder (ADHD),[4] obsessive-compulsive disorder (OCD),[5] Tourette’s disorder,[5] and schizophrenia,[6] among others. Understanding of association between familial autoimmunity and neuropsychiatric disorders can help in identifying subtypes of these disorders and guide treatment discovery.[7]

A limitation of interpreting studies that evaluate the association between family history of autoimmune disease and the risk of neuropsychiatric disorders in offspring is that the results may vary widely depending on the study design. For instance, the design of family studies published to date can be categorized as either registry-based[8–10] or questionnaire-based.[11–15] Both of these approaches have strengths and weaknesses. In registry-based studies, the family history of autoimmune disease is extracted from medical-record-based registries. Registry-based studies often have the advantage of large sample sizes. However, since autoimmune diseases are rare, identifying a sample of adequate size can be challenging even in large population studies, especially when the exposure or outcome being associated with autoimmune disease is also rare (i.e. particular neuropsychiatric disorders). In addition, diagnostic accuracy is often uncertain. Probands and family members are not readily available to query should clarification or additional information be needed about diagnoses in the registry. In many cases the presence or absence of standardized diagnostic codes in the records are used to determine diagnostic history,[8–10, 16, 17] a practice that can result in false positives. The presence of these codes does not necessarily establish a reliable history of the disorder or disease for the patient; codes may have been entered for reasons other than establishing the patient’s diagnosis, such as explaining the initial reason for a visit or obtaining insurance reimbursement. Furthermore, some patients may have been diagnosed based on clinical impression without verification by laboratory testing or consultation from a specialist. In addition to false positives, false negatives can result from patients receiving medical care outside of the system covered by the registry.

In questionnaire-based family studies, family history of autoimmune diseases is collected via questionnaires asking participants to self-report whether they and sometimes their relatives have been diagnosed with an autoimmune disease.[11–15] This design allows researchers to conduct a detailed examination of probands with neuropsychiatric disorders in order to establish valid clinical diagnoses and to further characterize phenotype. Although this study design gives investigators direct access to the patients and their relatives, the accuracy of self-reported autoimmune disease diagnoses has not typically been verified either by medical record review or by clinical assessment. In addition, using a questionnaire-based approach can introduce bias in risk estimates due to differential self-reporting of suspected risk factors among families of probands diagnosed with neuropsychiatric disorders.

The convenience and the potential pitfalls of the questionnaire-based design warrant investigation into the accuracy of self-report diagnosis. However, there are over 80 known autoimmune diseases affecting humans that vary widely in age of onset, prevalence, and severity.[18] Because of the low prevalence of the majority of autoimmune diseases, previous studies investigated the accuracy of self-reports for the few more common diseases. Several studies have evaluated the validity of self-reported diagnosis of certain autoimmune diseases, including hypothyroidism,[19, 20] diabetes mellitus (DM; including both type 1 and type 2),[21] rheumatoid arthritis (RA),[22–26] and psoriasis[27]. These studies typically determined accuracy of self-reports by reviewing the medical records of patients who reported a diagnosis. A limitation of this approach is that it cannot determine the number of individuals who have a diagnosis of autoimmune disease but do not report it (i.e. false negatives). In addition, these studies were often focused on the presence of one autoimmune disease, making it difficult to compare the accuracy of self-report across autoimmune diseases.

As part of a pilot study assessing the feasibility of using a self-report questionnaire to investigate history of autoimmune disease, we compared self-reported history of autoimmune disease in conjunction with electronic medical record (EMR) review for the 18 most common autoimmune diseases. This study was an initial step toward the longer-term goal of investigating the phenotypic correlates of family history of autoimmune disease in ASD. Medical record review is a commonly accepted way to validate self-reports,[19] but it is subject to challenges in physically obtaining a copy of medical records.[25, 28–30] To best overcome this challenge, we conducted this study at a large hospital-based adult primary care practice, which allowed for full access to EMR. With Institutional Review Board (IRB) approval and informed consent of individuals, we characterized the accuracy of self-report for the most prevalent autoimmune diseases using EMR-based diagnosis as the ‘gold standard’. Because this was a pilot study, we did not power it to demonstrate that the sensitivity and positive predictive value (PPV) of the questionnaire were above a given minimum threshold. However, we expected to obtain estimates consistent with 80% sensitivity and 80% positive predictive value (PPV) across conditions.

## Subjects and methods

This study was reviewed and approved by the Institutional Review Board of Partners HealthCare (Partners Human Research Committees) and all participants voluntarily provided written informed consent online prior to participation (protocol number 2013P001721).

### Subjects

Females and males between the ages of 18 and 70 years at the time of enrollment were included in the study. Patients whose primary care physician practiced at the Internal Medical Associates Primary Care Clinic at Massachusetts General Hospital (MGH) and who had an active account in the patient portal (a web-based system designed for patients to review their medical records and communicate with clinical providers) were identified through the Partners Healthcare Research Patient Data Registry. Thirty-one physicians of the practice were contacted, and 30 agreed to participate. Each physician reviewed the entire list of his or her patients and indicated which patients should be asked to participate in the study. Physicians excluded their patients based on personal judgment; potential reasons for exclusion were high patient burden, terminal illness, or other hardships. No other patients were excluded. The selected patients were contacted via an email signed by their physician describing the study and asking them to participate, with the option of signing an informed consent and a link to the secure online questionnaire. A total of 6220 patients of the 30 participating physicians were identified as eligible, and 5,999 (96% of those eligible) were asked to participate. Among those eligible 1,013 (17%) signed the informed consent and completed the questionnaire. Email invitations were sent between July 15, 2014, and February 10, 2015. Median time to survey completion was four days.

### Autoimmune history questionnaire

As part of the online autoimmune history questionnaire, patients were asked to report on a series of 33 autoimmune diseases (S1 Questionnaire). For each disease, patients answered the question “Have you or any of your relatives been told they have [***name of condition***]**?**” If patients responded affirmatively, they were asked to indicate the relative with the disease (including themselves), and the age range at which the relative with the disease was first told they had the disease, using a dropdown menu. Patients could choose to report on more than one relative for each disease. Patients also answered yes/no questions about autoimmune disease symptoms in the past three months and in their lifetime, questions about the numbers of biological relatives in their families, a question about history of other health conditions, a question about future participation in research, a question about family history of autism or developmental delay, a question about highest level of education, and an open-ended question allowing inclusion of additional information.

The autoimmune diseases of interest for this study were chosen based on their reported prevalence in the general population.[18] The chosen diseases, which had a population prevalence of 5 per 100,000 or higher, were Addison’s disease/ primary adrenocortical insufficiency, Graves’ disease, Hashimoto’s autoimmune thyroiditis, type 1 DM, celiac disease, Crohn’s disease, ulcerative colitis, alopecia areata, psoriasis, vitiligo, CREST syndrome, scleroderma, Sjögren’s syndrome, systemic lupus erythematosus (SLE), juvenile rheumatoid arthristis (JRA), RA, multiple sclerosis (MS), and myasthenia gravis (MG).

### Electronic medical record (EMR) review

The EMR for the 1,013 consented patients were extracted from the Research Patient Data Registry at Partners Healthcare and imported into a SQL Server 2016 database. EMR included clinical notes, problem lists, billing information, results of laboratory tests and procedures, and other information from the inpatient and outpatient encounters at MGH, authored by primary care physicians and other medical specialists. Clinical notes were reviewed if they were created within the time-frame of 5 years prior to and 18 months after the questionnaire was emailed. Detailed EMR review was conducted for each study participant if: 1) the problem list contained a diagnosis for any of the 18 autoimmune diseases of interest; or 2) the text extracted from the clinical notes indicated the possibility of a diagnosis for any of the 18 autoimmune diseases of interest. The text from the clinical notes was extracted using structured query language by matching keywords to the text in the clinical notes. These keywords included terminology that might be used to document a particular autoimmune disease diagnosis, symptoms that could precede the diagnosis of an autoimmune disease (S1 Table), and relevant laboratory/pathology tests (e.g., thyroid stimulating hormone (TSH) for hypothyroidism). These keywords were proposed by the study team and then edited by medical specialists, including primary care physicians, gastroenterologists, endocrinologists, dermatologists, neurologists, ophthalmologists, and rheumatologists, for accuracy and inclusiveness. Text surrounding these keywords (100 characters prior to the keyword and 100 characters after the keyword) was extracted together with the date and the author of the note. Clinical research staff reviewed the extracted text surrounding the keywords in order to exclude non-relevant information such as family history of autoimmune disease or an ongoing workup to rule out the diagnosis of an autoimmune disease. If the relevance of the extracted text was not clear, the clinical research staff read through the entire note or the whole record in the EMR for the patient. Although keyword searches were performed on all clinical notes in the EMR, a positive autoimmune disease diagnosis was given only if it was described in a clinical note authored by a physician. In addition, clinical research staff performed detailed EMR review for all individuals who self-reported presence of autoimmune disorders in the questionnaires. For any questionable diagnoses in the EMR, one of the physicians in the study reviewed the EMR to confirm diagnosis.

### Data analysis

Two by two tables were constructed for each of the 18 autoimmune diseases based on endorsement of the diagnosis by self-report and EMR review. Sensitivity, specificity, positive predictive value (PPV), and negative predictive value (NPV) were calculated for each autoimmune disease with an observed prevalence ≥ 0.5% by either self-report or EMR review. These calculations used the EMR diagnosis as the ‘gold standard’; i.e. the EMR diagnosis, but not necessarily the self-report diagnosis, was presumed to be accurate. Using type 1 DM as an example, sensitivity was calculated as the proportion of participants with type 1 DM, documented by EMR review, who also self-reported type 1 DM. Specificity was calculated as the proportion of participants without type 1 DM based on EMR review who did not self-report type 1 DM.[31] The PPV was calculated as the proportion of participants who self-reported type 1 DM who were also found to have type 1 DM by EMR review, i.e. the proportion of self-reported diagnoses confirmed by EMR review.[32] The NPV was calculated as the proportion of participants who did not self-report type 1 DM and did not have type 1 DM in the EMR review.[32] Data analysis, including calculation of exact binomial confidence intervals (CIs), was conducted using Stata Version 14.1 software (Stata Corp, College Station, TX). The power calculations for the discussion and the post-hoc chi-square test for trend assessing for association between education and diagnostic agreement were performed using the *power oneproportion* and *mhodds* commands respectively.

## Results

Physicians gave the study permission to contact 5,999 (96%) of 6,220 potential participants, of whom 1,013 (16% of potential participants and 17% of those contacted) provided informed consent to participate in the study and completed the questionnaire. Demographic characteristics of the respondents are reported in Table 1. Respondents had a mean (standard deviation) age of 52.6 (11.9) years, were 42.8% male, and were 92.7% white. Ninety-five percent of the participants had at least a two-year college degree.

**Table 1:** Demographics of 1,013 patients who provided informed consent and completed the autoimmune disease questionnaire.

Characteristics	N = 1,013
Age in years, Mean (St dev)	52.6 (11.9)
Sex, Number of Male (%)	434 (42.8%)
Education, Number (%)	
Some high school	3 (0.3%)
High school	40 (3.9%)
Some college or 2 year degree	138 (13.6%)
College graduate	348 (34.4%)
Advanced graduate or professional degree	477 (47.1%)
Missing	7 (0.7%)
Race/Ethnicity	
White	939 (92.7%)
Black	18 (1.8%)
Asian	30 (3.0%)
Hispanic	2 (0.2%)
Other or unknown	24 (2.4%)

Table 2 reports the frequency of autoimmune diseases (based on self-report and EMR review) and PPV (positive predictive value) by system. Table 3 reports estimates of sensitivity and PPV with associated exact binomial 95% confidence intervals for those diseases with an observed prevalence of at least 0.5% by self-report or EMR review. All specificity estimates for diseases of observed prevalence greater than 0.5% were greater than or equal to 98% with lower bound of the 95% CI greater than or equal to 97%. All estimates of NPV were greater than or equal to 96% with lower bound of the 95% CI greater or equal to 95%. Our focus is on estimation of sensitivity and PPV.

**Table 2:** Prevalence of autoimmune disease diagnosis according to self-report and medical record review, and overlap of self-report and medical record endorsement, by biological system.

	Self-reported Diagnosis, N (%^1^)	Medical Record Diagnosis, N (%^1^)	Self-reported Diagnosis Supported by Medical Record Diagnosis, N (PPV %^1^)
*Endocrine System*:			
Hashimoto's Thyroiditis	33 (3%)	17 (2%)	13 (39%)
Hypothyroidism^2^		108 (11%)	27 (82%)
Grave’s Disease	18 (2%)	15 (1%)	14 (78%)
Type 1 Diabetes Mellitus	12 (1%)	5 (0%)	5 (42%)
Addison’s Disease	1 (0%)	1 (0%)	0 (0%)
*Gastrointestinal System*:			
Celiac Disease	10 (1%)	11 (1%)	9 (90%)
Ulcerative Colitis	11 (1%)	10 (1%)	7 (64%)
Crohn’s Disease	4 (0%)	6 (1%)	4 (100%)
*Cutaneous System*:			
Psoriasis	23 (2%)	56 (6%)	17 (74%)
Alopecia	8 (1%)	23 (2%)	4 (50%)
Vitiligo	11 (1%)	4 (0%)	3 (27%)
*Systemic Autoimmune Diseases*:			
Sjögren’s Syndrome	7 (1%)	5 (0%)	5 (71%)
Systemic Lupus Erythematous	2 (0%)	3 (0%)	2 (100%)
CREST Syndrome	1 (0%)	1 (0%)	1 (100%)
Scleroderma	0 (0%)	0 (0%)	0 (0%)
*Musculoskeletal System*:			
Rheumatoid Arthritis	22 (2%)	13 (1%)	7 (32%)
Juvenile Rheumatoid Arthritis	2 (0%)	0 (0%)	0 (0%)
*Neurologic System*:			
Multiple Sclerosis	2 (0%)	2 (0%)	2 (100%)
Myasthenia Gravis	2 (0%)	2 (0%)	2 (100%)

1. Percentages with a self-reported and medical record diagnosis (columns 1 and 2) were calculated out of the total sample of 1,013 respondents. PPV (column 3) was calculated as a percentage out of the number of respondents with a medical record diagnosis in column 2.

2. Most medical record diagnoses of hypothyroidism did not specify if the disease was autoimmune in origin (i.e., Hashimoto’s thyroiditis). Though only 13/33 respondents who self-reported Hashimoto’s thyroiditis had the diagnosis of Hashimoto’s thyroiditis documented in the medical record (corresponding to a PPV of 39%), 27/33 had the diagnosis of hypothyroidism documented in the medical record (corresponding to a PPV of 82%).

**Table 3:** Estimated sensitivity and positive predictive value (95% confidence interval) for autoimmune diseases with minimum 0.5% prevalence by self-report or medical record review.

	Sensitivity (%)	Positive Predictive Value (%)
*Endocrine System*:		
Hashimoto's Thyroiditis^1^	76 (50, 93)	39 (23, 58)
Grave’s Disease	93 (68, 100)	78 (52, 94)
Type 1 Diabetes Mellitus	100 (48, 100)^2^	42 (15, 72)
*Gastrointestinal System*:		
Celiac Disease	82 (48, 98)	90 (55, 100)
Ulcerative Colitis	70 (35, 93)	64 (31, 89)
Crohn’s Disease	67 (22, 96)	100 (40, 100)^2^
*Cutaneous System*:		
Psoriasis	30 (19, 44)	74 (52, 90)
Alopecia	17 (5, 39)	50 (16, 84)
Vitiligo	75 (19, 99)	27 (6, 61)
*Systemic Autoimmune Diseases*:		
Sjögren’s Syndrome	100 (48, 100)^2^	71 (29, 96)
*Musculoskeletal System*:		
Rheumatoid Arthritis	54 (25, 81)	32 (14, 55)

1. Cases when a self-reported diagnosis of Hashimoto’s thyroiditis was reflected in a medical record diagnosis of hypothyroidism, but not Hashimoto’s thyroiditis, were considered negative for Hashimoto’s thyroiditis by medical record for these calculations.

2. One-sided 97.5% confidence interval.

### Endocrine system

The autoimmune diseases included from the endocrine system were Hashimoto’s thyroiditis, Grave’s disease, type 1 DM, and Addison’s disease. The most prevalent autoimmune disease of the endocrine system by both self-report (N = 33, 3%) and EMR review (N = 17, 2%) was Hashimoto’s thyroiditis. Of the 33 participants who self-reported a Hashimoto’s thyroiditis diagnosis and 17 participants who had the diagnosis according to EMR review, 13 had both a self-report and EMR diagnosis. The 13 with both a self-reported and EMR-based diagnosis out of 17 total participants with an EMR-based diagnosis corresponds to a sensitivity estimate of 76% (95% CI 50%:93%). In other words, we estimate that 76% of persons with a diagnosis of Hashimoto’s thyroiditis verified by EMR self-report the diagnosis. The 13 with both an EMR-based diagnosis and self-reported diagnosis out of 33 total participants with a self-reported diagnosis corresponds to a PPV estimate of 39% (95% CI 23%:58%). In other words, we estimate that 39% of persons who self-report a diagnosis of Hashimoto’s thyroiditis would have their diagnosis verified by EMR review.

The EMR review of the 20 participants who self-reported the diagnosis of Hashimoto’s thyroiditis but did not have the diagnosis of Hashimoto’s thyroiditis in their EMR (33 total self-reports minus 13 self-reports verified by EMR review) found that 14 of the participants had the diagnosis of “hypothyroidism.” In other words, more that 80% of patients who self-reported a diagnosis of Hashimoto’s thyroiditis had a diagnosis of hypothyroidism (with or without specified autoimmune origin) documented in their EMR. Of the remaining six participants who self-reported a diagnosis of Hashimoto’s thyroiditis that was not documented in their EMR, three had the diagnosis of “thyroid carcinoma,” one had the diagnosis of “Graves’ disease,” and two had no diagnosis indicative of thyroid abnormality documented. Further review of the EMR did not provide an explanation for the four participants with an EMR diagnosis of Hashimoto’s thyroiditis who did not endorse the diagnosis on the questionnaire.

The second most prevalent autoimmune disease of the endocrine system was Grave’s disease. Out of 18 participants who self-reported a Grave’s disease diagnosis and 15 participants with an EMR-based diagnosis 14 had both a self-report and EMR-based diagnosis. Out of the four participants with a self-reported diagnosis but no accompanying diagnosis in the EMR, three had a diagnosis of hypothyroidism, and one had no thyroid related diagnosis. The participant with the EMR diagnosis of Graves’ disease not reflected by self-report had not yet been diagnosed at the time of questionnaire distribution.

Twelve participants self-reported a diagnosis of type 1 DM, but only 5 participants had the diagnosis according to EMR review, all of whom were among the 12 endorsing the diagnosis by self-report. Of the seven participants with the self-reported diagnosis of type 1 DM but no accompanying diagnosis in the EMR, six had a diagnosis of type 2 DM in the EMR, and one had no related diagnosis.

The least prevalent autoimmune disease represented in the sample was Addison’s disease. One participant self-reported the diagnosis, and one different participant was diagnosed by EMR review. The participant with a self-reported diagnosis of Addison’s disease had the diagnosis of ‘left adrenal cortical adenoma, hyperaldosteronism (Conn's syndrome), adrenalectomy’ in the EMR. Further review of the EMR did not provide an explanation for the participant with an EMR diagnosis of Addison’s disease who did not endorse the diagnosis on the questionnaire.

#### Endocrine system: Hypothyroidism of unspecified etiology

The questionnaire asked about the presence of the diagnosis of Hashimoto’s thyroiditis without asking about the presence of the diagnosis of ‘hypothyroidism’. Because a high percentage of self-report diagnoses of Hashimoto’s thyroiditis not verified by EMR review had the diagnosis of ‘hypothyroidism’ in the EMR, and because we could not distinguish hypothyroidism of autoimmune origin from hypothyroidism of other etiologies, we recalculated our PPV estimate assuming self-report diagnoses of ‘Hashimoto’s thyroiditis’ accompanied by EMR diagnoses of ‘hypothyroidism’ were accurate. Using the EMR diagnosis of ‘hypothyroidism’ instead of the diagnosis of ‘Hashimoto’s thyroiditis’ to calculate the PPV of the self-reported diagnosis of Hashimoto’s thyroiditis increased the PPV estimate from 39% to 82%. We also reviewed the EMR of all patients for a diagnosis of hypothyroidism of any type and identified 108 participants with the diagnosis, among whom 17 (16%) had the diagnosis of Hashimoto’s thyroiditis and 91 (84%) had the diagnosis of “hypothyroidism” of unspecified etiology. Low prevalence of the Hashimoto’s thyroiditis diagnosis in the sample may reflect the absence of known etiology for many diagnoses of hypothyroidism, both for patients and medical professionals.

### Gastrointestinal system

The autoimmune diseases included from the gastrointestinal system were celiac disease, ulcerative colitis, and Crohn’s disease. Celiac disease and ulcerative colitis were more prevalent in the sample than Crohn’s disease. Out of ten participants who self-reported a diagnosis of celiac disease, and 11 participants with a diagnosis by EMR review, nine had both a self-report and EMR-based diagnosis. The participant who self-reported celiac disease without confirmation in the EMR was screened for the disease but had no diagnosis of celiac disease in the EMR. Further review of the EMR did not provide an explanation for the two participants with an EMR diagnosis of celiac disease who did not endorse the diagnosis on the questionnaire.

Out of 11 participants who self-reported a diagnosis of ulcerative colitis and 10 participants who had the diagnosis by EMR review, seven had both a self-report and EMR-based diagnosis. Out of the four participants who self-reported ulcerative colitis without an accompanying diagnosis in the EMR, two had a diagnosis of Crohn’s disease in the EMR, and two had undergone diagnostic colonoscopy, which had not resulted in a formal diagnosis at the time of the EMR review. Further review of the EMR did not provide an explanation for the three participants with an EMR diagnosis of ulcerative colitis who did not endorse the diagnosis on the questionnaire.

Four participants self-reported a diagnosis of Crohn’s disease, and six had the diagnosis by EMR review. All four participants who self-reported the diagnosis had the diagnosis reflected in their EMR. One out of the two participants with an EMR diagnosis of Crohn’s disease without the same self-reported diagnosis had a long-standing diagnosis of Crohn’s disease in the EMR without any discussion of the diagnosis or symptoms, probably indicating inactive symptoms.

### Cutaneous system

The autoimmune diseases included from the cutaneous system were psoriasis, alopecia, and vitiligo. These diseases were characterized by low estimated agreement between positive self-report and EMR-based diagnosis. Psoriasis was the most prevalent disease of the cutaneous system according to both self-report and EMR review, but was substantially more prevalent according to EMR review. Twenty-three participants self-reported a diagnosis of psoriasis, whereas 56 had the diagnosis according to EMR review. Seventeen of these participants had both a self-report and EMR diagnosis. All six participants with the self-report diagnosis of psoriasis not reflected in EMR were diagnosed with other dermatologic conditions (seborrheic keratosis; spongiotic dermatitis; alopecia areata; Raynaud’s disease, no ulcerations or lesions; dermatology consult, suspected psoriasis; and fungal infection). Of the 36 participants with the EMR diagnosis of psoriasis not reflected by the self-reported diagnosis of psoriasis, 29 were diagnosed by a dermatologist or a primary care physician, and the other seven had a history of the diagnosis documented in the primary care progress notes in the EMR. The fact that 29 participants diagnosed by a dermatologist or a primary care physician did not self-report the diagnosis might indicate that the diagnosis of psoriasis is self-reported during the acute phase of the disease but that having a history of this diagnosis is not self-reported.

Alopecia was also more prevalent by EMR review than by self-report. Eight participants self-reported the diagnosis, whereas 23 had the diagnosis according to EMR review. Only four of these participants had both a self-report and EMR-based diagnosis. Out of the four participants with the self-report diagnosis of alopecia unconfirmed by EMR, two had ‘skin dryness’, one had the diagnosis of vitiligo and one had no skin diagnoses. Out of the 19 participants with an EMR diagnosis not reflected by self-report, three recently received the diagnosis (possibly after completion of the questionnaire).

Vitiligo was more prevalent by self-report than EMR review. Eleven participants self-reported a diagnosis of vitiligo, whereas four participants had an EMR-based diagnosis. Three participants had a diagnosis both by self-report and EMR review. Out of the eight participants with the self-report diagnosis of vitiligo unconfirmed by EMR, one had the diagnosis of ‘possible vitiligo’, three had other skin conditions such as psoriasis, ‘actinic keratosis’, and skin rash, and four had no related diagnoses. Further review of the EMR did not provide an explanation for the participant with an EMR diagnosis of vitiligo who did not endorse the diagnosis on the questionnaire.

### Systemic autoimmune diseases

The systemic autoimmune diseases included were Sjögren’s syndrome, SLE, CREST syndrome, and scleroderma. Sjögren’s Syndrome was the only systemic autoimmune disease with observed prevalence of at least 0.5% by either self-report or EMR review. Seven participants self-reported the diagnosis, and five participants had the diagnosis according to EMR review. All five of the participants with the EMR-based diagnosis endorsed the disease by self-report. Of the two participants with the self-reported diagnosis of Sjögren’s syndrome, one had the diagnosis of ‘keratoconjunctivitis sicca’ in the EMR and the other had no related diagnoses.

Two participants self-reported a diagnosis of SLE, and three received the diagnosis by EMR review. EMR review supported the diagnosis for both participants who self-reported it. Further review of the EMR did not provide an explanation for the participant with an EMR diagnosis of SLE who did not endorse the diagnosis on the questionnaire. One patient had a diagnosis of CREST syndrome both according to self-report and EMR review. No participants had either a self-report or EMR-based diagnosis of scleroderma.

### Musculoskeletal system

The autoimmune diseases included from the musculoskeletal system were RA and JRA. RA was characterized by low estimates of agreement between self-report and EMR review. Twenty-two participants self-reported a diagnosis of RA, and 13 had the diagnosis according to EMR. Only seven of these participants had the diagnosis according to both self-report and EMR. Out of the 15 participants who self-reported a diagnosis of RA not reflected in the EMR, nine had the diagnosis of ‘osteoarthritis’, two had the diagnosis of ‘polymyalgia rheumatic’, two had the diagnosis of ‘back pain’, and two had no related diagnoses in the EMR. Of the six participants with an EMR diagnosis of RA not endorsed by self-report, one was seronegative for rheumatoid factor, and another had the diagnosis of RA as well as the diagnosis of osteoarthritis. Two participants self-reported a diagnosis of JRA, but no participants had a diagnosis of JRA according to EMR review. Of the two participants with a self-reported diagnosis of JRA, one had the diagnosis of RA and the other had no relevant diagnoses in the EMR.

### Neurologic system

The autoimmune diseases included from the neurologic system were MS and MG. Both had sample prevalence less than 0.5% by self-report and EMR review. The same two participants had a diagnosis of MS according to both self-report and EMR review. Another two participants had a diagnosis of MG according to both self-report and EMR review.

## Discussion

The integrity of research relying on self-reported diagnosis of personal and family history of autoimmune disease, including research associating family history of autoimmune disease with neuropsychiatric disorders,[33–35] and in our case ASD,[36, 37] depends on the accuracy of self-report. In this study, we compared the self-reported diagnosis of autoimmune disease with EMR-documented diagnosis of autoimmune disease in 1,013 adult patients receiving their medical care in a large hospital-based adult primary care practice, and found wide variability in the accuracy of self-reported diagnosis among the autoimmune diseases assessed. This study extends the literature by demonstrating considerable inaccuracy of self-report across different types of autoimmune diseases.

Unlike the majority of prior studies, this investigation reviewed diagnoses of autoimmune diseases identified both through self-report and EMR keyword searches, which allowed us to assess rates of both over-reporting and under-reporting. We first discuss the implications of our findings on accuracy for four representative autoimmune diseases with low estimates of sensitivity or PPV in our study: hypothyroidism, type 1 DM, RA, and psoriasis. We then discuss some key factors complicating the diagnosis of autoimmune diseases more generally.

### Hashimoto’s thyroiditis

The rate of hypothyroidism of any etiology based on EMR review in our study (10.7%) was higher than the rate for the U.S. population (4.6%)[38] possibly due to the high average age (53 years) and high proportion of individuals self-identifying with ‘white’ race (93%) in our sample. A U.S. study found mean values for TSH, which is typically elevated in patients with hypothyroidism, to increase with age for each decade following the twenties and to be higher for individuals identifying as ‘white, non-Hispanic’, than individuals of other races.[38] Hashimoto’s thyroiditis is the most common cause of hypothyroidism in the U.S., accounting for 90% of cases.[39]^,^[40] The proportion of cases of hypothyroidism in our sample (based on EMR review) diagnosed specifically with Hashimoto’s thyroiditis was 16% (17/108), a much lower proportion than would be expected if Hashimoto’s thyroiditis were routinely differentiated from other types of hypothyroidism in clinical practice. Lack of differentiation of Hashimoto’s thyroiditis from other types of thyroiditis may explain the low rate of Hashimoto’s thyroiditis by self-report (3%) and the low PPV for the self-reported diagnosis of Hashimoto’s thyroiditis in our study. Unfortunately, our questionnaire asked individuals to report only if they ever received a diagnosis of Hashimoto’s thyroiditis (without defining it as an autoimmune form of hypothyroidism), making it impossible to estimate the accuracy of self-report of hypothyroidism more generally.

Since the treatment of hypothyroidism does not require identification of a specific etiology, such as an autoimmune-mediated cause, it is likely that antibody tests required to differentiate autoimmune etiologies from other causes of hypothyroidism are not typically performed in a primary care setting. Our results indicate that neither self-report nor medical record review is sufficient for distinguishing between hypothyroidism with or without autoimmune origin; future studies requiring this distinction should consider validating the diagnosis with biological markers, such as testing for thyroid peroxidase antibodies.

### Type 1 DM

The present study observed over-reporting (PPV = 42%) but not under-reporting (sensitivity = 100%) of type 1 DM. Most false-positive self-reports were from participants with a diagnosis of type 2 DM. This type of over-reporting error is likely to occur in the presence of a related disease with a much greater prevalence. Type 1 DM is a relatively rare autoimmune disease while type 2 DM is a more common acquired disease with varying risk factors.[41] Type 2 DM is responsible for 90%–95% of DM cases,[41] with 8.3% of the U.S. population afflicted.[42] A tendency for a small percentage of persons with type 2 DM to misreport having type 1 DM could result in substantial inaccuracy of reports of type 1 DM.

Prior studies examining the accuracy of self-report of DM have reported PPV ranging from 64% to 98.5%,[43–50] with most studies reporting PPV above 90%. Unfortunately, these studies made no distinction in reporting accuracy between type 1 and type 2 DM. In a recent large population study of 266,848 individuals in Australia, 23,981 (9.0%) individuals reported an existing diagnosis of DM. In this study, the confirmation rate of self-reported DM (type 1 or type 2) by the hospital admission records was 79%.[21] However, when researchers evaluated the confirmation rate for type 1 DM, the PPV decreased to 42%. This suggests a much higher false positive rate for type 1 DM than DM of either type. Reviewing medication lists, asking about current and past insulin use, and asking about age of onset may improve the PPV of self-report measures for type 1 DM given the more frequent need for insulin therapy and lower age of onset for type 1 DM on average.[51] However, since the course and treatment for these two disorders overlap, and type 2 DM has much higher prevalence,[51, 52] it is unlikely that this would reduce the false positive rate substantially. Future studies should validate diagnosis of type 1 DM by laboratory testing and/or medical record review.

### Rheumatoid arthritis

The PPV of 32% obtained for RA in the present study is consistent with findings from previous studies reporting PPVs ranging from 19% to 34%.[16, 22–26] This study supported previous suggestions[16] that over-reporting of RA was mostly due to misreporting by individuals with other types of arthritis, such as osteoarthritis. Similar to over-reporting of type 1 DM by individuals with type 2 DM, the over-reporting of RA by individuals with osteoarthritis is due to the greater population prevalence of osteoarthritis (4.1% for the knee and 1.9% for the hip) than RA (0.4%).[53] In addition, accuracy of self-report of RA may depend on the questions asked and their context. Two large population-based studies asking about diagnosis of RA as part of extensive questionnaires about health outcomes and behaviors, the Nurse’s Health Study and the Iowa Women’s Health Study, were able to confirm fewer than 10% of self-reported diagnoses of RA.[25, 29] In contrast, several studies reported significant improvements in the accuracy of self-report by collecting information about drug regimen,[28, 30] asking about the presence of physical symptoms (using a validated questionnaire),[30] and inquiring whether the person is receiving medical care by a rheumatologist.[22] For example, a population study based on a subset of the Women’s Health Initiative cohort found that PPV increased from 15% to 62% when women brought in their medications to be recorded by research staff and information on the current medication was included in the analysis.[30] Together with existing research evidence, the low PPV and sensitivity (53%) observed in the current study suggest that future investigations should not rely on self-report diagnosis of RA without investigating and validating their method of assessment, using, for example, medical record review of a subset of cases.

### Psoriasis

Psoriasis is a common autoimmune skin disease with prevalence estimates for the general population ranging from 2 to 11%.[27, 54, 55] Our PPV estimate of 74% is close to the estimate of 78% found in a large population-based study in Norway that included a clinical examination by a dermatologist. However, our sensitivity estimate of 30% is lower than the same study’s estimate of 56%.[27] Our study found that some individuals did not self-report psoriasis even when moderate or severe forms of the disease were present in the EMR. It is possible that this could be due to the predominance of other serious medical concerns (such as cancer) complicating their clinical status. In fact, self-administered questionnaires are more sensitive for serious, life-threatening, acute-onset diseases[50] than for less serious diseases or diseases with intermittent appearance, especially diseases of skin.[56] It is also possible that there is diagnostic confusion from the individuals’ point of view (such as confusing the term ‘psoriasis’ with ‘eczema’, which we have seen in clinical practice) or poor communication between the health care provider and the patient due to ambiguous diagnostic criteria.[57] We would therefore recommend that researchers using self-report to diagnose psoriasis should be aware that a substantial percentage of cases may go unidentified.

Potential factors explaining variability in the accuracy of self-report of autoimmune diseases include the actual or potential impact of the diagnosis on the respondent and the complicated course of many autoimmune diseases. Differential significance attributed to past diagnoses can introduce bias in self-reports in family-based case control studies.[58]

Autoimmune diseases are heterogeneous disorders with varying ages of onset, which often wax and wane throughout a person’s lifetime. The age of onset, severity, and course of illness can be affected by pregnancy, aging, and stress. Some are more common in women than men. Some, like type 1 DM, typically have an age of onset in childhood, whereas others have a mean age of onset older than age 50.[18] Some, such as RA, SLE, and other autoimmune rheumatic diseases go through a preclinical disease phase lasting from months to several years characterized by little or no clinical findings but with the presence of detectable autoimmune antibodies.[59–64] Since the progression of autoimmune diseases can take years from the time that diagnostic markers of autoimmunity are present in the body to the time clinical symptoms appear and diagnosis occurs, it is possible that autoimmune diseases that will be diagnosed eventually will not become manifest clinically at the time a family study is conducted. In addition to the variable age of onset, an exacerbation of symptoms of some autoimmune diseases can occur during pregnancy and may go undetected in the absence of routine screening. For instance, thyroid disease affects 15% of pregnant women, but only about 20% of women are screened during pregnancy[65] and up to 2.5% of all pregnant women may have undiagnosed autoimmune connective tissue diseases.[66] Furthermore, some autoimmune diseases, such as thyroid diseases, appear during pregnancy, and then resolve after pregnancy.

### Limitations of this study

There are several limitations of this study’s findings. First, as reflected by the wide confidence intervals for our estimates, our preliminary study was not powered to demonstrate sensitivity or PPV above our chosen threshold of 80%. For example, achieving 80% power to demonstrate sensitivity or PPV above 80% for an individual disease would have required 113 self-reports or EMR reviews respectively for the disease, assuming an underlying population value of 90%. We observed a maximum of 33 self-reports (for Hashimoto’s thyroiditis) and 56 EMR-based diagnoses (for psoriasis). Unexpectedly, however, some of our observed sensitivity and PPV estimates were low enough that we could conclusively demonstrate sensitivity or PPV lower than 80%, results that demonstrate inadequate sensitivity or PPV in our setting. Although we cannot estimate sensitivity or PPV for any of the autoimmune diseases with precision, our results do provide evidence that the accuracy of self-reported diagnosis of autoimmune disease should not be assumed based on face validity. Based on the small numbers of participants we identified with autoimmune disease diagnoses, future studies may require the use of EMR or other databases accessing health information from large populations in order to achieve sample sizes sufficient for demonstrating the accuracy of alternative diagnostic strategies. In addition, future studies using similar methods of recruitment should allow for reductions in sample size due to physician exclusions and the requirement for allowing access to EMR. There is limited research available on how physician involvement in patient selection for EMR-based research studies affects patient recruitment. In our study, 4% of patients initially identified as eligible were excluded by their primary care physicians. Our completion rate of 17% is lower than the 30% response rate previously reported for questionnaires distributed through the patient portal,[67] a difference we attribute to asking permission for EMR access.

A second limitation is that the patients who completed the study questionnaire may not be representative of the general population due to differences between the population eligible for the study and the general population, response bias, or both. PPV and NPV depend on the prevalence of disease in the population, so our estimates would not directly generalize to populations with different rates of autoimmune disease. The study sample was substantially more educated and less racially diverse than the general population. Based on previous studies showing that advanced education significantly increases the accuracy of self-report of medical diseases,[50] we would expect based on educational attainment that our study population would self-report more accurately than the general population. A post hoc test for trend found no significant association between education level and any diagnostic disagreement between self-report and EMR review (odds ratio estimate per category increase in education level = 0.84, 95% CI 0.68: 1.04), though we note the low representation of persons without at least some college education in our sample. Our results may also not generalize to non-white, Hispanic, and more racially and ethnically diverse populations; and participating physicians may have excluded many patients experiencing acute illness and other hardships from the sample.

A third limitation is that we used diagnosis based on EMR review as the ‘gold standard’ with which to assess the accuracy of self-report diagnosis. Though we selected a patient population for whom we expected complete records and comprehensive medical histories, some patients may have had undiagnosed autoimmune disease or may have been misdiagnosed by their clinicians. We also assumed that our keyword search strategy identified all patients with the targeted diagnoses in their EMR. Fourth, the cross-sectional design of the study and low prevalence of autoimmune diseases in our sample prevented us from investigating the stability of accuracy of self-report over time and changes in the accuracy of self-report with age and time since diagnosis. Finally, we did not attempt to investigate the accuracy of questionnaire-based diagnoses of first and second-degree relatives, which have contributed to classification of autoimmune disease history in prior studies.

### Conclusions

The results of this study demonstrate that patients are not accurate reporters of their own history of some autoimmune diseases. Future studies investigating the association between autoimmune disease history and neuropsychiatric disorders, should be aware of the limitations of self-report as a method of diagnostic assessment. More broadly, physicians should be aware of the limitations of self-report when questioning their patients about family history of autoimmune diseases, particularly when the presence of a positive history would inform medical decision-making. More extensive questioning or review of medical records may be required to achieve adequate diagnostic accuracy.

## Supporting information

S1 Questionnaire Self-reported online questionnaire. (DOCX)

S1 Table Search terms for the 18 most prevalent autoimmune diseases. (DOCX)
