# Supplementary material for: Accuracy of self-reported history of autoimmune disease: A pilot study
Source: PLoS One. 2019 May 29;14(5):e0216526. doi: 10.1371/journal.pone.0216526 (PMC6541243; doi:10.1371/journal.pone.0216526)
Supplement: S1 Questionnaire — (DOCX) [file pone.0216526.s001.docx]

**S1 Questionnaire. Self-reported online questionnaire.**

|  |  | **During the past 3 months, have you:** |
| --- | --- | --- |
| A1 | □ Yes □ No | ...felt **pain in more than one joint**, not due to trauma or injury? |
| A2 | □ Yes □ No | ...noted **swelling in more than one joint**, not due to trauma or injury? |
| A3 | □ Yes □ No | ...felt **stiffness for at least an hour after you get up in the morning**? |
| A4 | □ Yes □ No | ...felt **weakness in your arms or legs**, even if you have not been very active? |
| A5 | □ Yes □ No | ...felt **frequent pins and needles sensations** in your arms, hands, legs, or feet? |
| A6 | □ Yes □ No | ...been **unsteady walking**? |
| A7 | □ Yes □ No | ...had a **tremor** (involuntary shaking of one or more body parts)? |
| A8 | □ Yes □ No | ...been **sweating more than you usually do**, not due to change in weather or exercise? |
| A9 | □ Yes □ No | ...noticed your **eyes are frequently red or painful**, not due to work or environmental reasons? |
| A10 | □ Yes □ No | ...noticed your **eyes frequently feel dry or burning**, not due to work or environmental reasons? |
| A11 | □ Yes □ No | ...noticed your **eyes seem to bulge or stick out more than they used to**, or do you feel you have a **more wide-eyed look** lately? |
| A12 | □ Yes □ No | ...had **diarrhea several times** or had **daily diarrhea for more than 10 days**, not related to an infection? |
| A13 | □ Yes □ No | ...had **blood in your stool**, not due to hemorrhoids? |
| A14 | □ Yes □ No | ...had **rectal pain**, not due to hemorrhoids? |
| A15 | □ Yes □ No | ...had **greasy, oily bowel movements**? |
| A16 | □ Yes □ No | ...been **unusually constipated**? |
| A17 | □ Yes □ No | ...had **stomach pain several times** or had **daily stomach pain for more than 10 days**, not related to an infection? |
| A18 | □ Yes □ No | ...noticed **white or brown patches on your skin**, not age spots or due to change in skincare products? |
| A19 | □ Yes □ No | ...noticed **rash on your face or other sun-exposed areas**, not due to a usual sunburn, change in weather, or skincare products? |
| A20 | □ Yes □ No | ...noticed your **skin is much dryer than usual**, not due to change in weather or skincare products? |
| A21 | □ Yes □ No | ...noticed **dry rash on your knees or elbows**, not due to change in weather or skincare products? |
| A22 | □ Yes □ No | ...noticed **loss of wrinkles on your face, hands or extremities,** or **unusual thickening or shininess of your skin**, not due physical labor, change in weather, or skincare products? |
| A23 | □ Yes □ No | ...developed **painful or open sores (ulcers) on your fingertips**? |
| A24 | □ Yes □ No | ...noticed the **skin on your fingers or face feels unusually tight**? |
| A25 | □ Yes □ No | ...developed **sores on your hands or feet**, not due to physical labor? |
| A26 | □ Yes □ No | ...developed **frequent sores inside your mouth** that are not known to be herpes? |
| A27 | □ Yes □ No | ...noticed **increased hair loss or clumps of hair falling out**? |
| A28 | □ Yes □ No | ...noticed your **voice has gotten deeper**? |
| A29 | □ Yes □ No | ...been **unusually thirsty**? |
| A30 | □ Yes □ No | ...been **urinating much more frequently or in larger amounts than usual**? |
| A31 | □ Yes □ No | ...**lost weight without trying to**? |
| A32 | □ Yes □ No | ...**lost more than 10 lbs** in the past 3 months? |
| A33 | □ Yes □ No | ...**gained weight without trying to**? |
| A34 | □ Yes □ No | ...**gained more than 10 lbs** in the past 3 months? |
| A35 | □ Yes □ No | ...noticed a **swelling in the front of your neck**? |
| A36 | □ Yes □ No | ...been **unusually sensitive to the heat**? |
| A37 | □ Yes □ No | ...been **unusually sensitive to the cold**? |
| A38 | □ Yes □ No | ...noticed your **hands turn white or blue and are painful or numb/tingly when exposed to cold**? |
| A39 | □ Yes □ No | ...noticed your **menstrual periods becoming much more irregular** than usual? (If not applicable, select "No".) |
|  |  | **Have you ever:** |
| A40 | □ Yes □ No | ...been **treated for a corneal ulcer on your eye**? |
| A41 | □ Yes □ No | ...had **complete loss of vision in one eye**, or **seen double for more than a couple days**? |
| A42 | □ Yes □ No | ...had **more than one miscarriage**? (If not applicable, select "No".) |
| A43 | □ Yes □ No | …had **fevers higher than 101°F or 38°C** for more than 3 days at a time without a known infection or occurring on a regular basis? |
| A44 | □ Yes □ No | …had **blood clots**, including deep venous thrombosis or pulmonary embolism? |

**Please answer these questions so we can make a family tree. Please count** **living as well as deceased relatives. We only need to know about your** **biological (i.e. "blood-related") relatives, not adopted relatives or relatives related to you by marriage.**

B1. How many **full sisters** do **you** have? ("full" meaning you share both biological parents) ^[[1]](#footnote-1)^

B2. How many **full brothers** do **you** have? ("full" meaning you share both biological parents) ^A^

B3. How many **half sisters** do **you** have? ("half" meaning you share only one biological parent) ^A^

B4. How many **half brothers** do **you** have? ("half" meaning you share only one biological parent) ^A^

B5. How many **daughters** do **you** have? ^A^

B6. How many **sons** do **you** have? ^A^

B8. How many **full brothers** does **your mother** have? (your **maternal uncles**) ^A^

B9. How many **full sisters** does **your father** have? (your **paternal aunts**) ^A^

B10. How many **full brothers** does **your father** have? (your **paternal uncles**) ^A^

B11. How many **daughters** do **your full siblings** have? (your **nieces**) ^A,^ ^[[2]](#footnote-2)^

B12. How many **sons** do **your full siblings** have? (your **nephews**) ^A, B^

**For the 33 autoimmune disorders listed below, please list yourself and any of your *living as well as deceased biological (i.e. "blood-related")* relatives who have been told by a doctor, nurse, or other health care professional that they have the disorder. Please select each relative's *relationship to you*, and the *age they were first told* that they have the disorder.**

**Do not worry if you've never heard of the disorder. Some of these disorders are very rare, so if you have not heard of it, it is most likely you do not have it.**

C1. Have you or any of your relatives been told they have**Multiple sclerosis? ^[[3]](#footnote-3)^**

*[If “Yes”] ^[[4]](#footnote-4)^*

| Yourself or Relatives with [disorder name]: | Age first told they have the disorder: |
| --- | --- |
| [dropdown] ^[[5]](#footnote-5)^ | [dropdown] ^[[6]](#footnote-6)^ |

C2. Have you or any of your relatives been told they have**Myasthenia gravis?**^C^

C3. Have you or any of your relatives been told they have**Narcolepsy?**^C^

C4. Have you or any of your relatives been told they have**Guillain-Barre syndrome?**^C^

C5. Have you or any of your relatives been told they have**Addison's disease /Primary adrenocortical insufficiency?**^C^

C6. Have you or any of your relatives been told they have **Type I Diabetes mellitus?**^C^

C7. Have you or any of your relatives been told they have**Graves' disease?**^C^

C8. Have you or any of your relatives been told they have**Hashimoto's autoimmune thyroiditis?**^C^

C9. Have you or any of your relatives been told they have**Autoimmune hepatitis type 1?**^C^

C10. Have you or any of your relatives been told they have**Celiac disease?**^C^

C11. Have you or any of your relatives been told they have**Crohn's disease?**^C^

C12. Have you or any of your relatives been told they have**Pernicious anemia/atrophic gastritis?**^C^

C13. Have you or any of your relatives been told they have**Primary biliary cirrhosis?**^C^

C14. Have you or any of your relatives been told they have**Primary sclerosing cholangitis?**^C^

C15. Have you or any of your relatives been told they have**Ulcerative colitis?**^C^

C16. Have you or any of your relatives been told they have**Antiphospholipid syndrome?**^C^

C17. Have you or any of your relatives been told they have**Immune thrombocytopenic purpura (ITP)?**^C^

C18. Have you or any of your relatives been told they have**Polymyositis /dermatomyositis?**^C^

C19. Have you or any of your relatives been told they have**Rheumatiod arthritis?**^C^

C20. Have you or any of your relatives been told they have**Juvenile arthritis?**^C^

C21. Have you or any of your relatives been told they have**Kawasaki disease?**^C^

C22. Have you or any of your relatives been told they have**Polyarteritis nodosa?**^C^

C23. Have you or any of your relatives been told they have **Rheumatic fever?**^C^

C24. Have you or any of your relatives been told they have**Temporal arteritis?**^C^

C25. Have you or any of your relatives been told they have**Alopecia areata?**^C^

C26. Have you or any of your relatives been told they have**Dermatitis herpetiformis?**^C^

C27. Have you or any of your relatives been told they have**Discoid lupus erythematosus?**^C^

C28. Have you or any of your relatives been told they have**Vitiligo?**^C^

C29. Have you or any of your relatives been told they have**Psoriasis vulgaris?**^C^

C30. Have you or any of your relatives been told they have**CREST syndrome?**^C^

C31. Have you or any of your relatives been told they have**Scleroderma?**^C^

C32. Have you or any of your relatives been told they have**Sjogren's syndrome?**^C^

C33. Have you or any of your relatives been told they have**Systemic Lupus Erythematosus?** ^C^

**If you have ever been told by a doctor, nurse, or other health care professional that you have any ongoing medical conditions – such as Hepatitis (Liver Disease), Type II Diabetes, Cancer, Stomach/Intestinal Disease, Kidney Disease, Heart Attack, Heart Failure, High Blood Pressure, Seizures, Stroke, Severe Headaches, or Lung Disease (like Asthma) – please list the condition(s), select the age you were first told you have the condition(s), and the current status of the condition(s).**

| Condition ^[[7]](#footnote-7)^ | Age first told you have the condition | Current Status |
| --- | --- | --- |
| [text] | [dropdown] ^F^ | [dropdown] ^[[8]](#footnote-8)^ |

E1. Would you like to be informed of other research opportunities available through the Lurie Center, part of Massachusetts General Hospital?

○ Yes ○ No

*[If “Yes”]* **What is the best way to let you know about these opportunities?**

**□ Phone *[If “Phone” checked]*** Best phone number to contact:

**□ Email *[If “Email” checked]*** Best email address to contact:

E2. Have you or any of your relatives been told they have **autism or developmental delay?**

○ Yes ○ No

*[If “Yes”]*

| Relatives with autism or developmental delay: | Age first told they have the disorder: |
| --- | --- |
| [dropdown] ^E^ | [dropdown] ^F^ |

E3. What is your highest level of education? ^[[9]](#footnote-9)^

E4. Is there anything else you would like us to know?

1. Dropdown options are: 0 | 1 | 2 | 3 | 4 | 5 | 6 | 7 | 8 | 9 | 10 [↑](#footnote-ref-1)
2. This item only appears if the answer to [B1] and/or [B2] are greater than 0. [↑](#footnote-ref-2)
3. Dropdown options are: Yes |No /Don’t know [↑](#footnote-ref-3)
4. For each disorder listed, table branches out if answer is Yes. Patient can enter as many relatives as needed. [↑](#footnote-ref-4)
5. Dropdown options are: Self |Mother |Father |Maternal Grandmother (your mother's mother) |Maternal Grandfather (your mother's father) |Paternal Grandmother (your father's mother) |Paternal Grandfather (your father's father) |Full Sister (you share both biological parents) |Full Brother (you share both biological parents) |Half Sister (you share only one biological parent) |Half Brother (you share only one biological parent) | Daughter |Son |Niece (your full sibling's daughter) |Nephew (your full sibling's son) |Maternal Aunt (your mother's full sister) |Maternal Uncle (your mother's full brother) |Paternal Aunt (your father's full sister) |Paternal Uncle (your father's full brother) [↑](#footnote-ref-5)
6. Dropdown options are: Pre-birth |Newborn |In Infancy |In Childhood |In Adolescence |20-29 years |30-39 years |40-49 years| 50-59 years |60 years or older | Unknown [↑](#footnote-ref-6)
7. Patient can add as many conditions as needed. [↑](#footnote-ref-7)
8. Dropdown options are: Still a problem | Well-Managed | Resolved [↑](#footnote-ref-8)
9. Dropdown options are: Eighth grade or less |Some high school |High school graduate |Some college / post-high school or 2 yr. degree |College graduate |Advanced graduate or professional degree [↑](#footnote-ref-9)
