# Supplementary material for: Accuracy of self-reported history of autoimmune disease: A pilot study
Source: PLoS One. 2019 May 29;14(5):e0216526. doi: 10.1371/journal.pone.0216526 (PMC6541243; doi:10.1371/journal.pone.0216526)
Supplement: S1 Table — (DOCX) [file pone.0216526.s002.docx]

**S1 Table. Search Terms for the 18 Most Prevalent Autoimmune Diseases.**

|  | **Keywords** | **Keywords for tests or medications** |
| --- | --- | --- |
|  |  |  |
| *Endocrine System:* |  |  |
| Addison’s Disease | Addison; Adrenal Crisis; Autoimmune Adrenalitis; Primary Adrenal Insufficiency | 21-Hydroxylase; ACTH Stimulation; P450c21; Salivary Cortisol; Serum Cortisol; Urine Cortisol |
| Grave’s Disease | Grave | TBII, TSH; Free T4; T3; TBII, Thyrotropin-binding inhibiting;  TRAb, Thyroid Receptor Antibodies; Thyroid-Stimulating Immunoglobulin |
| Hashimoto's Thyroiditis | Autoimmune Thyroiditis; Hashimoto’s; Hashimotos; Hypothyroidism | TSH; Free T4; T3; Levothyroxine; Thyr; Thyroglobulin, Tg; TPO, Thyroid peroxidase; Thyrotropin |
| Hypothyroidism | Hypothyroidism | TSH; Free T4; T3; Levothyroxine; Thyr; Thyroglobulin, Tg; TPO, Thyroid peroxidase; Thyrotropin |
| Type 1 Diabetes Mellitus | Diabetes Mellitus Type 1; DM 1; Type 1 Diabetes; Insulin Dependent Diabetes; Juvenile-Onset Diabetes | C-Peptide; Fasting Insulin |
|  |  |  |
| *Gastrointestinal System:* |  |  |
| Celiac Disease | Celiac | Small Bowel Biopsy; Anti-DGP; IgA TTG, Anti-Tissue Transglutaminase; Duodenal Biopsy; IgA endomysial  HLA DQ2/DQ8 |
| Crohn’s Disease | Crohn | Colonoscopy; Sigmoidoscopy |
| Ulcerative Colitis | Ulcerative Colitis | Colonoscopy; Sigmoidoscopy |
|  |  |  |
| *Cutaneous System:* |  |  |
| Alopecia | Alopecia |  |
| Psoriasis | Psoriasis | Skin Biopsy |
| Vitiligo | Vitiligo | Wood’s Lamp |
|  |  |  |
| *Systemic System:* |  |  |
| CREST Syndrome | CREST Syndrome; Calcinosis; Raynaud; Esophageal Dysmotility; Sclerodactyly; Telangiectasias |  |
| Scleroderma | Scleroderma; Sclerodactyly; Skin Thickening | ACA, Anti-centromere; Anti-RNA Polymerase; Anti-Scl-70; Anti-Topoisomerase |
| Sjögren’s Syndrome | Sjogren |  |
| Systemic Lupus Erythematous | Lupus | ANA, Anti-Nuclear Antibody; PTT-LA |
|  |  |  |
| *Musculoskeletal System:* |  |  |
| Juvenile Rheumatoid Arthritis | Juvenile Rheumatoid Arthritis; Juvenile Arthritis; Juvenile Idiopathic Arthritis; Still’s Disease |  |
| Rheumatoid Arthritis | Rheumatoid Arthritis | Rheumatoid Factor; ANTI-CCP, Anti-Cyclic Citrullinated Peptide Antibodies |
|  |  |  |
| *Neurologic System:* |  |  |
| Multiple Sclerosis | Multiple Sclerosis; Demyelination | Brain MRI; Periventricular; Oligoclonal Bands, Oligoclonal IGG Bands |
| Myasthenia Gravis | Myasthenia Gravis; Seronegative Myasthenia; Edrophonium | AChR; EMG, Electromyography; Ice Pack Test; MuSK; RNS; Tensilon Test |
